# Dictionary of Elevations of the United States

**Published:** 1873-12-15

**Authors:** 


"DICTIONARY OF ELEVATIONS OF THE UNITED STATES."—We understand that Dr. J. M. Toner, of Washington, D. C., has a work in press with the foregoing title. We have before us proof-sheets of the first eight or ten pages. From the matter contained in these sheets, it is evident that the work will be one of great value to all who wish to study medical topography and climate in connection with vital statistics. For instance, one of the tables before us gives the average elevation above the sea-level of each State and Territory, with the number of square miles, the number of persons to the square mile, the total population, and the proportion of population living in cities of over 5,000 inhabitants. Another gives the average altitude of each State and Territory, in connection with the percentage of deaths to the population in 1850, 1860 and 1870; the percentage of deaths from consumption to the total mortality in 1860 and 1870; the percentage of deaths from diseases of the nervous system in 1870; and the percentage of deaths by diseases of the respiratory system to the total mortality for 1870. In another table we find a list of all the cities of over 5,000 inhabitants, in the United States, with their altitude, and population according to the census of 1870. We hope the work will be speedily issued from the press, and liberally patronized by the profession.

